# Amphibians of the equatorial seasonally dry forests of Ecuador and Peru

**DOI:** 10.3897/zookeys.1063.69580

**Published:** 2021-10-18

**Authors:** Diego Armijos-Ojeda, Diana Székely, Paul Székely, Dan Cogălniceanu, Diego F. Cisneros-Heredia, Leonardo Ordóñez-Delgado, Adrián Escudero, Carlos Iván Espinosa

**Affiliations:** 1 Laboratorio de Ecología Tropical y Servicios Ecosistémicos (EcoSs-Lab), Departamento de Ciencias Biológicas y Agropecuarias, Universidad Técnica Particular de Loja, Loja 110107, Ecuador; 2 Programa de Doctorado en Conservación de Recursos Naturales, Escuela Internacional de Doctorado, Universidad Rey Juan Carlos, 28933 Móstoles, Madrid, Spain; 3 Museo de Zoología, Universidad Técnica Particular de Loja, San Cayetano Alto, calle París s/n, Loja, Ecuador; 4 Faculty of Natural and Agricultural Sciences, Ovidius University Constanţa, 900470, Constanţa, Romania; 5 Colegio de Ciencias Biológicas y Ambientales COCIBA, Universidad San Francisco de Quito USFQ, Quito 170901, Ecuador; 6 Museo de Zoología & Laboratorio de Zoología Terrestre, Instituto de Biodiversidad Tropical iBIOTROP, Universidad San Francisco de Quito USFQ, Quito, Ecuador; 7 Department of Geography, King’s College, London, UK; 8 Instituto Nacional de Biodiversidad INABIO, Quito, Ecuador; 9 Department of Science, Rey Juan Carlos University, 28933, Móstoles, Madrid, Spain

**Keywords:** Annotated list, Anura, Conservation, Distribution, Herpetofauna, Life-history

## Abstract

Seasonally dry forests (SDFs) are one of the most challenging ecosystems for amphibians, fueling the diversity of this group of vertebrates. An updated inventory of native amphibians present in the Equatorial SDF is provided, which extends along the Pacific coast of Ecuador and northwestern Peru. The study is based on an extensive field sampling (two thirds of the total records) carried out throughout the Equatorial SDF, along with a compilation of the available information on distribution of amphibians in the region from published scientific papers, museum collections and on-line databases. The final dataset included 2,032 occurrence records for 30 amphibian species, belonging to eight anuran families. Additionally, data regarding conservation status, habitat use, spawn deposition site, reproductive mode, and body size, along with an identification key for all encountered species are provided. The results indicate a strong sampling bias with a deficit in the Peruvian part of the study area, and a need for urgent inventories targeted at under-sampled areas, using modern taxonomic methods. The study emphasizes the conservation priorities in the Equatorial SDF, based on the distribution, conservation status and life-history data. This information should be useful for the local authorities and institutions involved in the management and conservation of biodiversity in SDF.

## Introduction

Seasonally dry forests (hereafter SDFs) have been recently recognized as a coherent biome distributed across South America (Prado and Gibbs 1993; Pennington et al. 2000; Pennington et al. 2006; Linares-Palomino et al. 2011). They consist of tree- or shrub-dominated ecosystems with deciduous or semideciduous vegetation, occurring in frost-free areas with mean annual temperatures higher than 17 °C, high seasonal rainfall that sums less than 1,600 mm/year, and at least 5–6 months annually with less than 100 mm/month (Murphy and Lugo 1986; Pennington et al. 2000; Prado 2000; Espinosa et al. 2012). Although animal diversity of Neotropical SDFs has received relatively little attention (Sánchez‐Azofeifa et al. 2005), a general trend of lower species richness is apparent when compared to neighboring moister forest ecosystems such as rainforests and cloud forests (Espinosa et al. 2011; Hanson 2011; Jenkins et al. 2013; Guedes et al. 2018). This trend is quite evident in amphibians, organisms that are highly dependent of humid conditions. The harsher climate conditions typical for the SDF act as strong limiting factors for amphibian diversity (Duellman 1988; Székely et al. 2016). Even so, survey efforts carried out in these habitats have revealed high levels of amphibian endemism, and diverse behavioral and physiological adaptations allowing most of these species to endure long periods of low food availability and hydric stress (Ceballos 1995; Chazdon et al. 2011; Stoner and Timm 2011).

In the Neotropics, there are at least four distinct phytogeographic groups of SDF: Caribbean-Mesoamerican, Ecuadorian-Peruvian, Brazilian Caatinga, and Central South American (Prado 2000; Linares-Palomino 2004a). Among them, the Ecuadorian-Peruvian SDF has the smallest extent, aggregating coastal SDFs from western Ecuador and northwestern Peru (Pennington et al. 2000; Peralvo et al. 2007), but excluding the seasonal habitats from Huancabamba and Marañon, which, although relatively close spatially, are considered to be biogeographically distinct due to the fact that the Andes mountain-range represents a dispersal barrier (Linares-Palomino 2004b). Chapman (1926) was the first to recognize the high levels of biodiversity and endemism of the Ecuadorian SDF, using the term Tropical Arid Fauna. Later, the name Tumbesian Centre of Endemism has been extensively used (Cracraft 1985; Best and Kessler 1995; Stattersfield et al. 1998) for this biogeographic region, recognized as a center of endemism at a global scale taking into consideration the better studied taxa, i.e., birds (Best and Kessler 1995) and vascular plants (Davis et al. 1997) and, consequently, a global priority for conservation (DryFlor 2016) and a hotspot for biodiversity (Myers et al. 2000). Other authors have referred to this area under different (complete or partially synonym) names: Ecuadorian Subcentre (Müller 1973), Guayas Province (Ringuelet 1975), Ecuadorian Pacific Dry Forest (Udvardy 1975), Pacific Equatorial Dominion (Ab’Saber 1977), Tumbesian Centre (Cracraft 1985), Ecuadorian Dry Forest and Western Ecuador Moist Forest (Dinnerstein 1995), Western Ecuador Province (Morrone 1999), Arid Ecuadorian and Túmbes-Piura Provinces (Morrone 2001), Equatorial Pacific Area (Porzecanski and Cracraft 2005), Western Ecuador and Ecuadorian Provinces (Morrone 2014), and there is currently a lack of consensus about the precise position and extent of the SDF in Ecuador and Peru. These diverse definitions are usually based on endemism patterns of either vascular plants or birds, so they tend to include neighboring moist habitats, ranging from mangroves to montane cloud forests (Best and Kessler 1995), merging different ecosystems which are often not characterized by seasonality. As a result, these delimitations are less effective when applied to more water-dependent taxa such as amphibians, which show quite different patterns of diversity and endemism.

The amphibian diversity in the SDF of the coastal areas of Ecuador and Peru has been scarcely explored, with only a small number of localities being inventoried (Almendáriz and Carr 1992, 2012; Venegas 2005; Cisneros-Heredia 2006; Armijos-Ojeda and Valarezo 2010; Amador and Martínez 2011; Székely et al. 2016; Sánchez-Nivicela et al. 2015; Cuadrado et al. 2020). Several factors influence this lack of information, including bias caused by researchers’ preference for the more biodiverse tropical rain and cloud forests, logistic limitations imposed by site accessibility, and the short and unpredictable rainy season when amphibians are active and can be detected.

The first step in the development of any effective management and conservation strategy for amphibians is the completion of regional inventories, especially in the context of rapid biodiversity loss and climate changes. Understanding species distribution is especially urgent in the case of amphibians, the most threatened vertebrate group worldwide (Catenazzi 2015). In this context, our aim was to update the list of amphibian species and their distribution in the coastal SDF of Ecuador and Peru, through extensive fieldwork and the compilation of all available information, to prioritize conservation actions, promote public awareness and focus further inventory efforts towards areas where gaps remain.

## Materials and methods

### Study area

For the purpose of the study, we use the definition of the Ecuadorian Province (Morrone 2014), including all seasonally dry forests (**SDFs**) in this biogeographical region and excluding neighboring moist habitats that are likely to promote amphibian communities of different origin and with different characteristics. Henceforth, we will use the term Equatorial SDF for this area, which has a finer resolution than the one of Ecuadorian Province; also, we consider the term to be more adequate to denominate territories in both Ecuador and Peru. To generate the map layer used in the analysis, we used Quantum GIS (QGIS) environment 3.4.13 (QGIS.org 2021). To delimit our study area, we used as a basis the national digital maps of ecosystem types for Ecuador (MAE 2013) and Peru (MINAM 2019). These two cartographic databases are currently the most precise available for the area, due to their spatial resolution (scale 1:100,000). In both cases, the ministries of environment in the respective countries define the types of ecosystems according to vegetation cover, bioclimate, biogeography, physiography, altitude, and land use cover. The final map for the Equatorial SDF included ecosystem types with a characteristic of seasonal distribution of precipitation and a semi-deciduous and deciduous vegetation (forests, shrublands), and excluded the Marañón dry forests (Suppl. material 1: Table S1). We added the “Anthropical” and “No data” categories situated in areas of historical distribution of those ecosystems. The resulting shape was manually corrected, fixing geometry problems and filling gaps with the dedicated tool of QGIS to reduce the noise and obtain a more accurate area. The final area covers 55,680.5 km^2^ (of which 36.5% in Ecuador and 63.5% in Peru), with an altitudinal range between 0 and 1631 m a.s.l., and consists of a narrow band (3–150 km wide) bordering the Pacific Ocean, extending from the Ecuadorian province of Esmeraldas in the north, to the Peruvian department of Lambayeque in the south.

The climate in the Equatorial SDF region is characterized by a striking seasonality, with a dry season lasting between five and eight months (Escribano-Ávila 2016), a fairly stable high temperature throughout the year, and annual rainfall varying between 500–1,500 mm, while the average monthly rainfall varies between 10 mm to more than 200 mm (Murphy and Lugo 1986; Espinosa et al. 2012). The vegetation is dominated (>50%) by deciduous or semi-deciduous trees. The region is delimited by neighboring ecosystems characterized by a higher rainfall input, such as the transition zones to the Andean mountain range (foothills) in the eastern region and transition zones to the Choco rainforest in the north.

### Data collection

**Figure 1.**
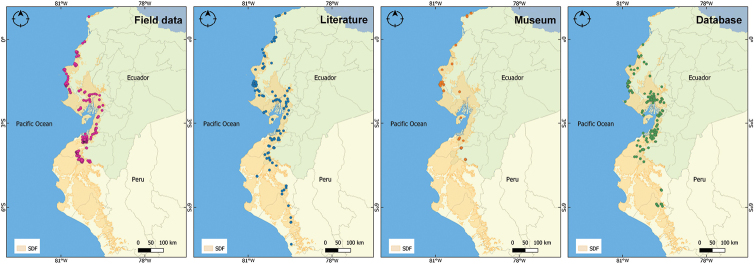
Distribution of amphibian occurrence records in the Equatorial Seasonally Dry Forest (SDF). Maps are provided depending on the data source: Field data, Literature, Museum, Database.

The distribution records were compiled from the following sources:

1. Field surveys. Field data were collected and geo-referenced by the authors between 2000 and 2021. Sampling was carried out at various locations (Fig. 1 – Field data), using visual / auditory encounter surveys and active searches (Heyer et al. 1994). Specific methodologies varied, but consisted in both diurnal and nocturnal extensive surveys carried out mainly during the rainy season, and included searches of suitable terrestrial refugia, netting, torching, pitfall traps and call surveys, unconstrained by time or area.
2. Literature review. We carried out search routines between January and April 2021 on the online search engines Google Scholar (https://scholar.google.com/), ScieLO (https://scielo.org/), Web of Science (http://webofknowledge.com/), retrieving papers by using the following search terms: “amphibian”, “Anura”, “herpetofauna”, and “Tumbesian”, “Ecuadorian dry forest”, “Peruvian dry forest”, and reviewing the first 200 results for each search. We included articles in peer-reviewed journals, as well as theses and reports that included relevant information regarding the species distribution, where locations were either geo-referenced or precise enough to permit the assignation of coordinates, and identification was done to species level (Fig. 1 – Literature).
3. Museum biological collections housed at Instituto Nacional de Biodiversidad, Quito, Ecuador (DHMECN), and Museo de Zoología, Universidad San Francisco de Quito, Ecuador (ZSFQ).
4. Publicly available species distribution data on the Global Biodiversity Information Facility (https://www.gbif.org/), which includes the iNaturalist platform data, accessed April 2021 (https://doi.org/10.15468/dl.55dnar). These data were manually curated, removing all vague locality descriptors, likely erroneous species identification, and exotic species records (Zizka et al. 2020). We also filtered for duplicated records (same species at the same coordinates at the same moment).

Regardless of source, we standardized the species list using the taxonomy of Amphibian Species of the World (Frost 2021). Only specimens that could be identified to species level were included in the dataset. Non-native species records were removed (i.e., the bullfrog *Lithobatescatesbeianus*). For each species, we indicate the extinction risk status at the global level based on the IUCN Red List of Threatened Species (IUCN 2021).

To characterize species life-history traits, we carried out a literature search for each species in peer-reviewed articles or books and completed with field observations whenever available (Suppl. material 2). We selected four relevant traits which reflect ecological strategies, niche, and functional roles in the ecosystem (Oliveira et al. 2017) and adopted some rather coarse categories to accommodate for the lack of ecological information for most of the species present in the region. Species habitat use, defined as the overall vertical foraging stratum preferred by the adult, resulted in four broad categories: terrestrial/fossorial (foraging mostly on the ground or in leaf-litter, galleries, crevices, or holes on the floor), arboreal (predominantly perching on leaves in trees, bushes, phytotelmata, grasses, including riparian vegetation), terrestrial/riparian (found in terrestrial habitats close to or around bodies of water), and aquatic/riparian (semi-aquatic species living in streams or ponds). We also reported the reproductive mode (either larval or direct development), as well as the spawn site, the microhabitat where eggs are deposited (either aquatic, terrestrial, or arboreal). As a morphological character, body size was defined as the maximum snout-vent length (**SVL**) value known for the species, and we report the value separately for females and males. Finally, we generated an identification key, based on morphological characters. However, it is worth mentioning that in some taxa (e.g., the case of *Engystomops* spp.) the reliable identification usually requires additional information (such as mating calls).

### Specimen collection

In the case of voucher specimens, individuals were photographed, after which they were euthanized using 20% benzocaine, fixed in 10% formalin, and stored in 70% ethanol. Tissue samples for genetic analyses were preserved in 96% ethanol. Specimens are deposited at Museo de Zoología, Universidad Técnica Particular de Loja (MUTPL), and Museo de Zoología, Universidad San Francisco de Quito (ZSFQ) Ecuador. Information on these specimens is included as field data since it was generated by the authors during fieldwork.

Research permits were issued by Ministerio del Ambiente del Ecuador. This study was evaluated and approved by the Ethics Committee of Universidad Técnica Particular de Loja (UTPL-CBEA-2016-001).

## Results

The final dataset consists of 2,032 distribution records spread throughout the Equatorial SDF region. Seventy-seven records are from Peru, and 1,955 are from Ecuador (Fig. 1). Our field records constitute most of the data points (Fig. 1 – Field data), i.e. 1,374 records (67.6%). The literature revision produced 285 records (14%) from 29 publications (Fig. 1 – Literature), while the museum collections of INABIO and ZSFQ included 87 records (4.3%, Fig. 1 – Museum). The online databases GBIF and iNaturalist contributed 286 data points, representing 14.1% of the dataset (Fig. 1 – Database).

**Figure 2.**
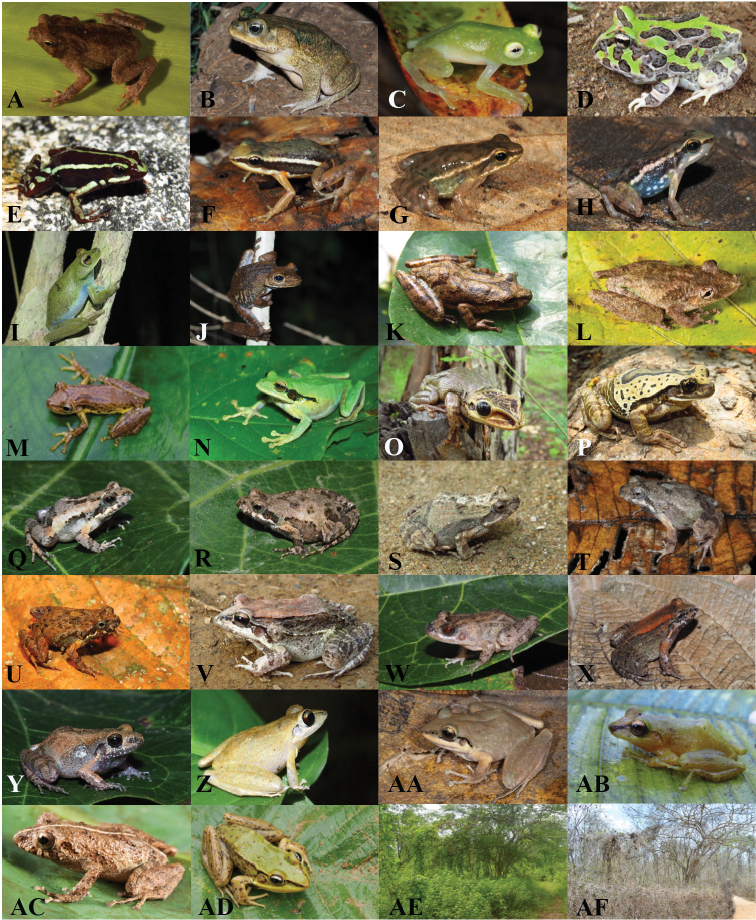
Amphibian species of the Equatorial Seasonally Dry Forest **A***Rhinellaalata* (photo by Silvia Aldás, https://bioweb.bio) **B***Rhinellahorribilis***C***Hyalinobatrachiumtatayoi***D***Ceratophrysstolzmanni***E***Epipedobatesanthonyi***F***Epipedobatesmachalilla***G***Hyloxaluselachyhistus***H***Hyloxalusinfraguttatus***I***Boanapellucens***J***Boanarosenbergi***K***Scinaxquinquefasciatus***L***Scinaxsugillatus* (photograph by Santiago R. Ron, https://bioweb.bio) **M***Scinaxtsachila***N***Smiliscaphaeota***O***Trachycephalusjordani***P***Trachycephalusquadrangulum***Q***Engystomopsguayaco***R***Engystomopsmontubio***S***Engystomopspustulatus***T***Engystomopspuyango***U***Engystomopsrandi***V***Leptodactyluslabrosus***W***Leptodactylusmelanonotus***X***Leptodactylusventrimaculatus***Y***Barycholospulcher***Z***Pristimantisachatinus***AA***Pristimantislymani***AB***Pristimantissubsigillatus***AC***Pristimantiswalkeri* (photograph by Santiago R. Ron, https://bioweb.bio) **AD***Lithobatesbwana*. Habitat seasonal change (Reserva Ecológica Arenillas) **AE** april (rainy season) **AF** december (dry season).

**Figure 3.**
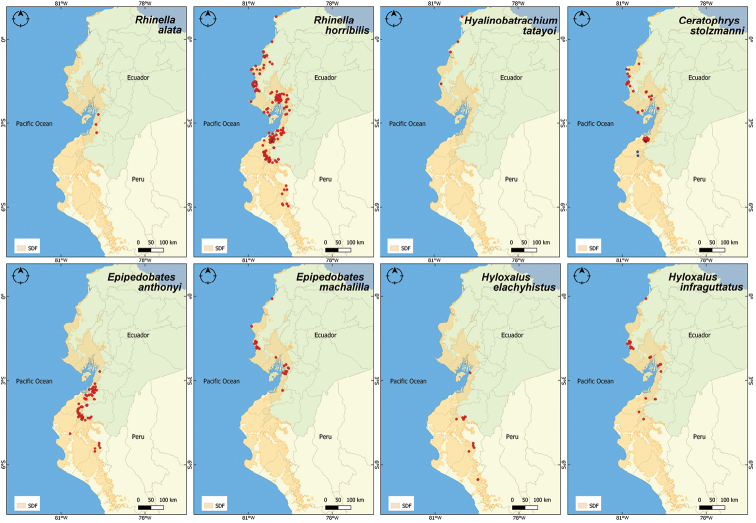
Distribution records of Bufonidae, Centrolenidae, Ceratophryidae and Dendrobatidae in the Equatorial Seasonally Dry Forest (SDF). Maps are given for the families Bufonidae (*Rhinellaalata*, *R.horribilis*), Centrolenidae (*Hyalinobatrachiumtatayoi*), Ceratophryidae (*Ceratophrysstolzmanni*) and Dendrobatidae (*Epipedobatesanthonyi*, *E.machalilla*, *Hyloxaluselachyhistus*, *H.infraguttatus*). For *Ceratophrysstolzmanni*, blue points represent new distributional records for the species, the two southernmost localities and the highest altitude, respectively.

**Figure 4.**
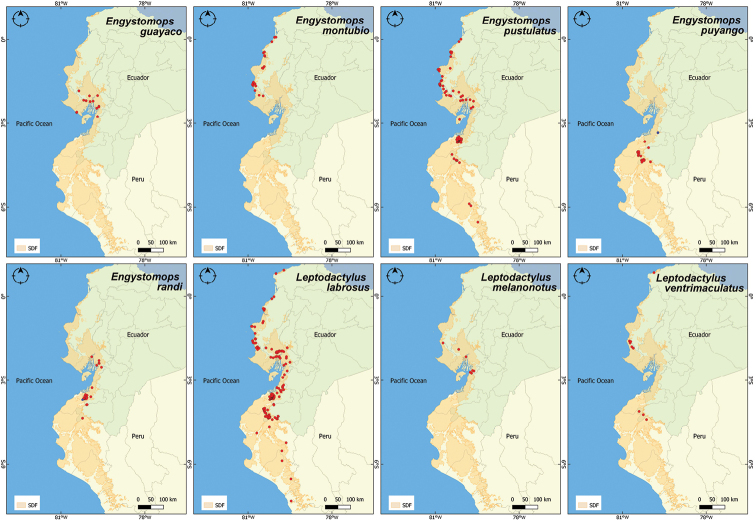
Distribution records for the Leptodactylidae family in the Equatorial Seasonally Dry Forest (SDF). In blue, distribution range extensions: for *Engystomopspuyango*, the northernmost locality is more than 70 km from the previously known distribution; for *E.randi*, the first record in Peru.

**Figure 5.**
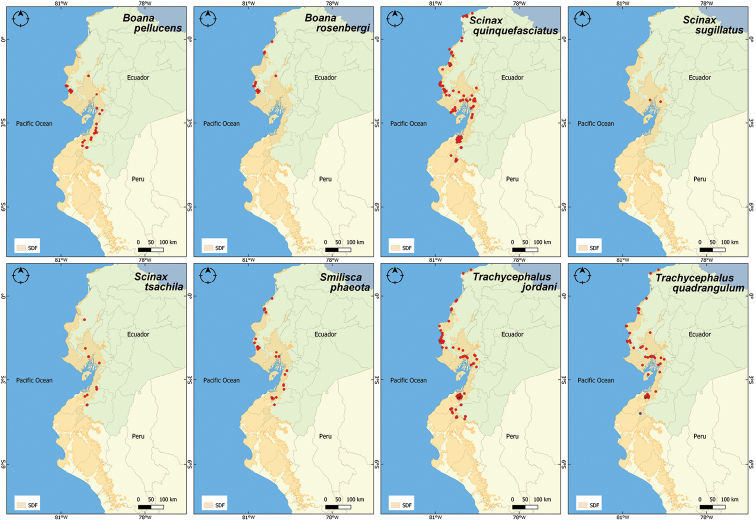
Distribution records for the Hylidae family in the Equatorial Seasonally Dry Forest (SDF). In blue, the first report of *Trachycephalusquadrangulum* in Loja province.

**Figure 6.**
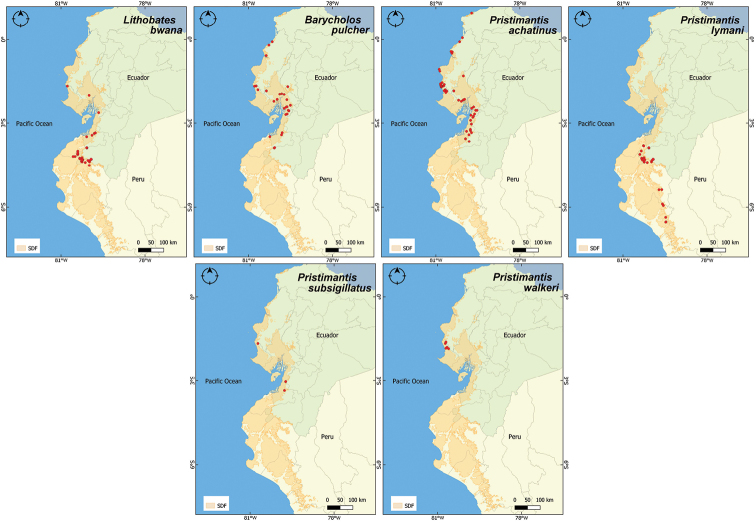
Distribution records of Ranidae and Craugastoridae in the Equatorial Seasonally Dry Forest (SDF). Maps are given for Ranidae (*Lithobatesbwana*) and Craugastoridae (*Barycholospulcher*, *Pristimantisachatinus*, *P.lymani*, *P.subsigillatus*, and *P.walkeri*).

Overall, we report 30 amphibian species for the Equatorial SDF, belonging to 14 genera and eight families (Figs 2–6); all 30 species were present in Ecuador, of which 16 were also encountered in Peru (Suppl. material 1: Table 2S). The best represented family was Leptodactylidae (genera *Engystomops* and *Leptodactylus*) with eight species. Five species (*Ceratophrysstolzmanni*, *Engystomopsmontubio*, *E.puyango*, *E.randi* and *Lithobatesbwana*) are endemic to the Equatorial SDF. Two, *Epipedobatesanthonyi* and *Leptodactyluslabrosus*, have a distribution mostly restricted to the Equatorial SDF, with few occurrences in adjacent habitats, characterized by higher humidity/altitude. The remaining 23 species have a wider distribution.

Regarding the global extinction risk status (IUCN 2021), one (*C.stolzmanni*) is classified as Vulnerable, and three are Near Threatened (*E.anthonyi*, *Hyalinobatrachiumtatayoi*, and *Hyloxalusinfraguttatus*). Two are Data Deficient (*Rhinellaalata* and *Engystomopsguayaco*) and another three (*R.horribilis*, *Scinaxtsachila* and *Trachycephalusquadrangulum*) are currently Not Evaluated, while the remaining 21 species have a Least Concern IUCN status (Table 1).

**Table 1.** Life-history characteristics and conservation status for the amphibians of the Equatorial Seasonally Dry Forest. IUCN Status – extinction risk status according to IUCN (2021): NE - Not Evaluated, DD - Data Deficient, LC - Least Concern, NT - Near Threatened, VU - Vulnerable. Reproductive modes: LDv - Larval Development, DDv - Direct Development. * indicates species with a distribution restricted to Equatorial Seasonally Dry Forest. References are given in Suppl. material 2. FD - unpublished information collected by the authors during fieldwork.

Family	Species	IUCN Status	Habitat	Spawn site	Reproductive mode	Maximum size (males) mm	Maximum size (females) mm	References
Bufonidae	*Rhinellaalata*	DD	Terrestrial / fossorial	Aquatic	LDv	43.3	56.2	FD; (1)
	*Rhinellahorribilis*	NE	Terrestrial / fossorial	Aquatic	LDv	130.0	160.0	FD; (2); (3); (4)
Centrolenidae	*Hyalinobatrachiumtatayoi*	NT	Arboreal	Arboreal	LDv	26.8	31.1	FD; (5); (6)
Ceratophryidae	Ceratophrysstolzmanni*	VU	Terrestrial / fossorial	Aquatic	LDv	70.4	75.9	FD; (7); (8); (9)
Dendrobatidae	*Epipedobatesanthonyi*	NT	Terrestrial / fossorial	Terrestrial	LDv	25.0	27.0	FD; (10); (11)
	*Epipedobatesmachalilla*	LC	Terrestrial / fossorial	Terrestrial	LDv	16.0	17.6	FD; (12); (13)
	*Hyloxaluselachyhistus*	LC	Aquatic / riparian	Terrestrial	LDv	24.1	24.8	FD; (12); (14)
	*Hyloxalusinfraguttatus*	NT	Terrestrial / fossorial	Terrestrial	LDv	20.5	23.4	FD; (12); (15); (16)
Hylidae	*Boanapellucens*	LC	Arboreal	Aquatic	LDv	52.9	61.0	(17); (18); (19); (20); (21); (22)
	*Boanarosenbergi*	LC	Arboreal	Aquatic	LDv	90.0	93.2	(19); (23); (24); (25)
Hylidae	*Scinaxquinquefasciatus*	LC	Arboreal	Aquatic	LDv	38.2	38.9	(26); (27)
	*Scinaxsugillatus*	LC	Arboreal	Aquatic	LDv	42.0	45.5	(27); (28)
	*Scinaxtsachila*	NE	Arboreal	Aquatic	LDv	34.2	36.4	FD
	*Smiliscaphaeota*	LC	Arboreal	Aquatic	LDv	66.0	78.0	(29)
	*Trachycephalusjordani*	LC	Arboreal	Aquatic	LDv	95.4	111.3	FD; (28); (30)
	*Trachycephalusquadrangulum*	NE	Arboreal	Aquatic	LDv	76.9	80.8	FD; (28); (31)
Leptodactylidae	*Engystomopsguayaco*	DD	Terrestrial / fossorial	Aquatic	LDv	19.38	20.98	FD
	Engystomopsmontubio*	LC	Terrestrial / fossorial	Aquatic	LDv	22.8	19.71	FD
	*Engystomopspustulatus*	LC	Terrestrial / fossorial	Aquatic	LDv	32.3	36.5	FD
	Engystomopspuyango*	LC	Terrestrial / fossorial	Aquatic	LDv	30.5	32.6	FD; (13); (32)
	Engystomopsrandi*	LC	Terrestrial / fossorial	Aquatic	LDv	18.7	19.7	(13); (33)
	*Leptodactyluslabrosus*	LC	Terrestrial / fossorial	Terrestrial	LDv	67.4	71.2	FD; (34); (35); (36)
	*Leptodactylusmelanonotus*	LC	Terrestrial / riparian	Terrestrial	LDv	43.4	48.1	(35); (37); (38)
	*Leptodactylusventrimaculatus*	LC	Terrestrial / riparian	Terrestrial	LDv	55.4	59.3	FD
Strabomantidae	*Barycholospulcher*	LC	Terrestrial / fossorial	Terrestrial	DDv	26.9	30.5	(39); (40)
	*Pristimantisachatinus*	LC	Terrestrial / fossorial	Terrestrial	DDv	36.2	46.1	(19); (41); (42)
	*Pristimantislymani*	LC	Terrestrial / fossorial	Terrestrial	DDv	45.3	72.9	FD; (43); (44)
	*Pristimantissubsigillatus*	LC	Terrestrial / fossorial	Terrestrial	DDv	28.5	33.4	FD; (45)
	*Pristimantiswalkeri*	LC	Terrestrial / fossorial	Terrestrial	DDv	18.5	25.3	FD
Ranidae	Lithobatesbwana*	LC	Aquatic / riparian	Aquatic	LDv	63	95	FD; (46)

### Life-history characteristics

In terms of amphibian species habitat use, 17 (56.7%) are terrestrial / fossorial, nine (30%) are arboreal, two are aquatic / riparian (6.7%), and two are terrestrial / riparian (6.7%) (Table 1). Most species have larval development (25 species, 83.3%), and the five species of Strabomantidae have a direct development (16.7%). Amphibians living in Equatorial SDF exhibit several reproductive strategies for egg deposition; the most common behavior was to deposit eggs directly in the water (17 species, 56.6%, amongst which the five *Engystomops* species which produce foam nests), terrestrial deposition (12 species, 40%), and one species lays egg clutches on leaves overhanging water (*Hyalinobatrachiumtatayoi*). The range of body sizes is wide, with maximum adult size varying between 16 mm (*Epipedobatesmachalilla*) and 130 mm (*Rhinellahorribilis*).

### Changes in distribution range

We report here the extension of the distribution ranges of four amphibian species detected during fieldwork.

*Ceratophrysstolzmanni* (Pacific horned frog). This species is endemic to the lowland Equatorial SDF (Ortega-Andrade et al. 2021), with a distribution extending from its type locality, Tumbes, Peru (Steindachner 1882), in the south, up to La Seca (Manabí, Ecuador), in the north. Distribution follows the Pacific coast, the innermost point being 50 km from the coast (Cuadrado et al. 2020), but all previously recorded locations were at low altitudes (up to 130 m a.s.l.). In the present study, we extend the known distribution of this species by adding several new locations (Fig. 3). Amongst them, the record from Manabí, Ecuador (1.0679°S, 80.8308°W), in the vicinity of the El Aromo oil refinery, at 380 m a.s.l., is the highest altitude reported for the species. We also encountered the Pacific horned frog in Progreso, Reserva Cazaderos (4.0259°S, 80.4497°W, 221 m a.s.l.) and Mangahurco, Área de Conservación Municipal Los Guayacanes (4.1611°S, 80.4388°W, 360 m a.s.l.), these being the first records for the Loja province (Ecuador). They also represent the most continental records for this species, being located at more than 70 km from the Pacific coast. Another important observation is that the locations in Loja province, despite being spatially close to the Tumbes region, are actually separated by the Cerro de Amotape mountain range, which was until now considered a barrier for this typically lowland, burrowing amphibian.

*Engystomopspuyango* (Puyango dwarf frog). This small amphibian was recently described from the Puyango Petrified Forest, in south-western Ecuador (Ron et al. 2014), and was until now known from a small number of localities. We contribute several new reports in the region; its presence in Casacay (3.3383°S, 79.7268°W, 146 m a.s.l.), El Oro province, more than 72 km from the type locality, constitutes the farthest record from the known distribution (Fig. 4).

*Engystomopsrandi* (Rand’s dwarf frog). Another recently described leptodactylid species, which has a wider distribution, encompassing most of the Equatorial SDF close to the coast (Ron et al. 2014). We report for the first time its presence in Peru, Tumbes Reserve (3.7743°S, 80.2249°W, 53 m a.s.l.) (Fig. 4).

*Trachycephalusquadrangulum* (Chocoan milk frog). This is a large tree frog, mostly known from the coastal Ecuadorian region (Ron et al. 2016). We contribute a new locality for Loja province in Ecuador, close to Bolaspamba (4.1823°S, 80.3692°W, 416 m a.s.l.) (Fig. 5).

### Key to the amphibian species of the Equatorial seasonally dry forests of Ecuador and Peru

**Table:** 

1	Digit tips not expanded	**2**
–	Digit tips expanded	**13**
2	Keratinized metatarsal spade present; extremely wide head and mouth	***Ceratophrysstolzmanni***
–	Keratinized metatarsal spade absent	**3**
3	Parotoid glands present	**4**
–	Parotoid glands absent	**10**
4	Cranial crests present; adults medium or large: SVL > 40 mm; flank glands absent	**5**
–	Cranial crests absent; adults small: SVL < 40 mm; flank glands present	**6**
5	Large sized, SVL of adults > 70 mm; parotoid glands large; tarsal fold present	***Rhinellahorribilis***
–	Medium sized, SVL of adults < 60 mm; parotoid glands small; tarsal fold absent	***Rhinellaalata***
6	SVL of adults > 23 mm; lateral fringes on toes absent	**7**
–	SVL of adults < 23 mm; lateral fringes on toes present	**8**
7	SVL of adults > 25 mm; larger tubercles on the dorsum	***Engystomopspustulatus***
–	SVL of adults > 23 mm; smaller and fewer tubercles on the dorsum	***Engystomopspuyango***
8	SVL of adults 15–20 mm; lateral fringes on toes broad; webbing between toes basal	***Engystomopsguayaco***
–	Lateral fringes on toes narrow, webbing between toes absent	**9**
9	SVL of adults 17–22 mm; proportionately shorter flank and parotoid glands	***Engystomopsmontubio***
–	SVL of adults 17–20 mm; proportionately longer flank and parotoid glands	***Engystomopsrandi***
10	Extensive webbing between the toes; subarticular tubercles low	***Lithobatesbwana***
–	Webbing between the toes absent; subarticular tubercles well developed	**11**
11	Males with black horny thumb spines; toes with well-developed lateral fringes	***Leptodactylusmelanonotus***
–	Males without thumb spines; toes without developed lateral fringes	**12**
12	Posterior surface of tarsus with many white tubercles; sole of foot with white tubercles	***Leptodactylusventrimaculatus***
–	Posterior surface of tarsus usually without white tubercles; sole of foot usually lacking white tubercles	***Leptodactyluslabrosus***
13	Expanded discs bearing a pair of scute-like fleshy structures on the dorsal surface of digit tips	**14**
–	Expanded discs without dorsal scute-like fleshy structures on tips of digits	**17**
14	Broad, light middorsal stripe present	***Epipedobatesanthonyi***
–	Middorsal stripe absent	**15**
15	Venter immaculate (without white spots)	***Epipedobatesmachalilla***
–	Venter with white spots	**16**
16	Extensive webbing between the toes	***Hyloxaluselachyhistus***
–	Limited webbing between the toes	***Hyloxalusinfraguttatus***
17	Venter transparent with the white peritonea and lungs visible, dorsal surfaces green with yellow spots	***Hyalinobatrachiumtatayoi***
–	Venter not transparent and internal organs not visible, dorsal surfaces brown, grey or green	**18**
18	Fingers lacking webbing	**19**
–	Webbing present between fingers	**26**
19	Toe III longer than Toe V; digit tips just slightly expanded (swollen); well defined white glands posterior to angle of jaw	***Barycholospulcher***
–	Toe V longer than Toe III	**20**
20	Toes lacking extensive webbing	**21**
–	Webbing present between toes	**24**
21	Finger I longer than Finger II; dorsolateral folds present	**22**
–	Finger I shorter than Finger II; dorsolateral folds absent	**23**
22	Discs on fingers relatively small; inner surface of tarsus bearing long fold; posterior surfaces of the thighs black with white spots or reticulations; SVL of adults 25–73 mm	***Pristimantislymani***
–	Discs on fingers broad; inner tarsal tubercle small; posterior surfaces of the thighs brown with small cream flecks; SVL of adults 23–46 mm	***Pristimantisachatinus***
23	Snout bearing papilla at tip; heel with small conical tubercle; SVL of adults 19–33 mm	***Pristimantissubsigillatus***
–	Snout without papilla at tip; heel lacking tubercles; groin black with yellow spots; SVL of adults 13–25 mm	***Pristimantiswalkeri***
24	Lower jaw with a row of tubercles; snout long; black and blue mottling in the groin and on the anterior and posterior surfaces of the thighs	***Scinaxsugillatus***
–	Lower jaw without a row of tubercles	**25**
25	Shank bones visible through the skin, white to bluish-white; dorsum with scattered to abundant small tubercles	***Scinaxquinquefasciatus***
–	Shank bones visible through the skin, green; dorsum without tubercles	***Scinaxtsachila***
26	Top of the head co-ossified and rough (integumentary-cranial co-ossified skull); iris golden with irregular black spots; SVL of adults 65–111 mm	***Trachycephalusjordani***
–	Top of the head not co-ossified	**27**
27	Skin on dorsum tuberculate; webbing between the fingers extensive; dorsal coloration usually brown	***Boanarosenbergi***
–	Skin on dorsum smooth; webbing between fingers basal to moderate	**28**
28	Pronounced calcar on the heel; webbing between the fingers moderate; dorsal coloration usually green; iris yellowish	***Boanapellucens***
–	Calcar on heel absent	**29**
29	Webbing between the fingers moderate; iris golden with irregular black spots; thick, glandular skin on the head and back	***Trachycephalusquadrangulum***
–	Webbing between the fingers basal; characteristic dark postorbital mark and white labial stripe	***Smiliscaphaeota***

### Clave para las especies de anfibios del bosque estacionalmente seco Ecuatorial de Ecuador y Perú

**Table:** 

1	Terminaciones de los dedos no expandidas	**2**
–	Terminación de los dedos expandidas	**13**
2	Presencia de espádices metatarsiales queratinizados; cabeza y boca extremadamente anchas	***Ceratophrysstolzmanni***
–	Espádice metatarsial queratinizado ausente	**3**
3	Presencia de glándulas parotoideas	**4**
–	Glándulas parotoideas ausentes	**10**
4	Presencia de crestas craneales; adultos medianos o grandes: LHC > 40 mm; glándulas del flanco ausentes	**5**
–	Crestas craneales ausentes; adultos pequeños: LHC < 40 mm; glándulas del flanco presentes	**6**
5	Tamaño grande, LHC de adultos > 70 mm; glándulas parotoideas grandes; pliegue tarsal presente	***Rhinellahorribilis***
–	Tamaño mediano, LHC de adultos < 60 mm; glándulas parótidas pequeñas; pliegue tarsal ausente	***Rhinellaalata***
6	LHC de adultos > 23 mm; flecos laterales en los dedos de los pies ausentes	**7**
–	LHC de adultos < 23 mm; flecos laterales en los dedos de los pies presentes	**8**
7	LHC de adultos > 25 mm; tubérculos más grandes en el dorso	***Engystomopspustulatus***
–	LHC de adultos > 23 mm; menos tubérculos y de tamaño menor en el dorso	***Engystomopspuyango***
8	LHC de adultos de 15 a 20 mm; flecos laterales en los dedos de los pies anchas; membrana entre los dedos de los pies basal	***Engystomopsguayaco***
–	flecos laterales en los dedos del pie estrechos; membranas entre los dedos del pie ausentes	**9**
9	LHC de adultos de 17 a 22 mm; glándulas parotoideas y del flanco proporcionalmente más pequeñas	***Engystomopsmontubio***
–	LHC de adultos de 17 a 20 mm; glándulas parotoideas y del flanco proporcionalmente más largas	***Engystomopsrandi***
10	Extensas membranas entre los dedos de los pies; tubérculos subarticulares bajos	***Lithobatesbwana***
–	Membranas entre los dedos de los pies ausentes; tubérculos subarticulares bien desarrollados	**11**
11	Machos con espinas córneas negras en los pulgares; dedos de los pies con flecos laterales bien desarrollados	***Leptodactylusmelanonotus***
–	Machos sin espinas pulgares; dedos de los pies sin flecos laterales desarrollados	**12**
12	Superficie posterior del tarso con muchos tubérculos blancos; planta del pie con tubérculos blancos	***Leptodactylusventrimaculatus***
–	Superficie posterior del tarso generalmente sin tubérculos blancos; planta del pie generalmente sin tubérculos blancos	***Leptodactyluslabrosus***
13	Discos expandidos que llevan un par de estructuras carnosas en forma de escudos en la superficie dorsal de las puntas de los dedos	**14**
–	Discos expandidos sin estructuras carnosas en forma de escudos dorsales en las puntas de los dedos	**17**
14	Presencia de una franja media dorsal clara y ancha	***Epipedobatesanthonyi***
–	Franja media dorsal ausente	**15**
15	Vientre inmaculado (sin manchas blancas)	***Epipedobatesmachalilla***
–	Vientre con manchas blancas	**16**
16	Membrana extensa entre los dedos de los pies	***Hyloxaluselachyhistus***
–	Membrana limitada entre los dedos	***Hyloxalusinfraguttatus***
17	Vientre transparente con el peritoneo blanco y los pulmones visibles, superficies dorsales verdes con manchas amarillas	***Hyalinobatrachiumtatayoi***
–	Vientre no transparente y órganos internos no visibles, superficies dorsales marrón, gris o verde	**18**
18	Dedos de la mano sin membranas interdigitales	**19**
–	Membranas interdigitales presentes entre los dedos de la mano	**26**
19	Dedo III del pie más largo que el Dedo V; puntas de los dedos solo ligeramente expandidas (hinchadas); glándulas blancas bien definidas posteriores al ángulo de la mandíbula	***Barycholospulcher***
–	Dedo V del pie más largo que el Dedo III	**20**
20	Dedos del pie que carecen de membranas extensas	**21**
–	Membranas interdigitales presentes entre los dedos de los pies	**24**
21	Dedo I del mano más largo que el Dedo II; pliegues dorsolaterales presentes	**22**
–	Dedo I del mano más corto que el Dedo II; pliegues dorsolaterales ausentes	**23**
22	Discos en los dedos relativamente pequeños; superficie interna del tarso con pliegue largo; superficies posteriores de los muslos negras con manchas o reticulaciones blancas; LHC de adultos 25–73 mm	***Pristimantislymani***
–	Discos en los dedos anchos; tubérculo tarsal interno pequeño; superficies posteriores de los muslos marrones con pequeñas manchas color crema; LHC de adultos 23–46 mm	***Pristimantisachatinus***
23	Hocico con papila en la punta; talón con pequeño tubérculo cónico; LHC de adultos 19–33 mm	***Pristimantissubsigillatus***
–	Hocico sin papila en la punta; talón sin tubérculos; ingle negra con manchas amarillas; LHC de adultos 13–25 mm	***Pristimantiswalkeri***
24	Mandíbula inferior con una hilera de tubérculos; hocico largo; moteado negro y azul en la ingle y en las superficies anterior y posterior de los muslos	***Scinaxsugillatus***
–	Mandíbula inferior sin una hilera de tubérculos	**25**
25	Huesos de las patas visibles a través de la piel, de color blanco a blanco azulado; dorso con pequeños tubérculos, dispersos a abundantes	***Scinaxquinquefasciatus***
–	Huesos de las patas visibles a través de la piel, verdes; dorso sin tubérculos	***Scinaxtsachila***
26	Parte superior de la cabeza co-osificada y rugosa (cráneo co-osificado tegumentario-craneal); iris dorado con manchas negras irregulares; LHC de adultos 65–111 mm	***Trachycephalusjordani***
–	Parte superior de la cabeza no co-osificada	**27**
27	Piel en el dorso tuberculada; membrana extensa entre los dedos de la mano; coloración dorsal generalmente marrón	***Boanarosenbergi***
–	Piel lisa en el dorso; membrana entre los dedos basal a moderada	**28**
28	Calcar pronunciado en el talón; membrana entre los dedos de la mano moderada; coloración dorsal generalmente verde; iris amarillento	***Boanapellucens***
–	Calcar en el talón ausente	**29**
29	Membrana entre los dedos de la mano moderada; iris dorado con manchas negras irregulares; piel glandular gruesa en la cabeza y dorso	***Trachycephalusquadrangulum***
–	Membrana entre los dedos de la mano basal; marca postorbital oscura característica y franja labial blanca	***Smiliscaphaeota***

## Discussion

We provide the first comprehensive amphibian species checklist for the Equatorial SDF, including 30 species. In addition to compiling the available data from published sources, museum collections and online databases, we contribute a large amount of original information generated through extensive field surveys (two thirds of all reported information for the area). Although the records reported here significantly add to our previous understanding of tropical amphibian communities in South American seasonally dry habitats, the dataset probably underestimates the actual amphibian diversity in the area.

Although the Equatorial SDF has been overall understudied, the lack of information is most evident in the Peruvian part of this ecoregion. For a better understanding, further efforts to disseminate currently unpublished amphibian distribution records of Peruvian researchers and taxonomically clarify the identity of amphibians which are currently assigned only at genus level (e.g., Venegas 2005) are necessary. From the total dataset, less than 4% of the records were from Peru, although 63.5% of Equatorial SDF area corresponds to this country. A lower amphibian richness is expected in certain Peruvian regions, such as the area bordering the Sechuran desert, due to the hostile environmental conditions. The bibliographic search and a comparison with similar habitats in Ecuador suggest that the lack of data regarding amphibian diversity in the Peruvian part of the Equatorial SDF is due to sampling bias rather than accurately reflecting the absence of this taxa. Even in Ecuador, where sampling was carried out more homogenously throughout the study area, there is still a shortage of adequate amphibian inventories, especially outside protected areas (Ortega-Andrade et al. 2021). Further efforts to inventory the extensive underexplored areas to correctly evaluate the amphibian community status should constitute a priority (Ortega-Andrade et al. 2021).

The fact that, out of the 30 species present in the Equatorial SDF, five have been described as new for science in the last 20 years (*Scinaxtsachila*, *Engystomopsguayaco*, *E.montubio*, *E.puyango*, *E.randi*) further emphasizes the need for intense and focused research targeted at undersampled locations. The list of amphibians present in the Equatorial SDF can change in the future because of updated taxonomic studies based on modern integrative techniques that use morphological, molecular, and behavioral data. It is the case of the cane toads (*R.horribilis*), for which a recent study indicates that the species present in these forests might be phylogenetically distinct from the rest of the range (Pereyra et al. 2021). Similarly, a species of milk frog (*Trachycephalusquadrangulum*) was resurrected after being included in the *T.typhonius* species complex for 50 years, as was the toad *Rhinellaalata*, after being synonymized to *R.margaritifera*. It is likely that a similar fate awaits species in the genera *Pristimantis*, *Leptodactylus* and *Hyloxalus*, for which taxonomical delimitation is currently based on morphological characters only, allowing for the existence of cryptic taxa.

We include the information on important life history characteristics for all amphibian species present in the Equatorial SDF. It is recommended that prioritization of conservation measures should consider functional diversity of an assemblage, not only species richness, since species make differential contribution to the functioning of their ecosystem (Campos et al. 2017; Bolochio et al. 2020). Currently, research conducted on life-history is scarce for most of the 30 amphibian species. As more information becomes available, the inclusion of additional traits, might offer a more complete image of the native amphibian communities and their capacity to withstand landscape changes. The current insufficient knowledge regarding Equatorial SDF species threats and risks, in addition to the fact that some have been only recently (re)described, results in the five species that are lacking a global conservation status assessment.

Seven of the 30 species (23.3%) have a distribution exclusively or almost-exclusively restricted to the Equatorial SDF. Although amphibian species living in tropical dry forests are inherently more tolerant to high temperatures and desiccation, they are still expected to be vulnerable to the predicted climate changes because they are already exposed to conditions at the limit of their physiological tolerance (Catenazzi et al. 2014; Székely et al. 2018). No studies modelling the sensitivity to climate change scenarios have been carried out for the species endemic to the Equatorial SDF. Some of the species have adapted to anthropized environments, and in some cases their distribution extends to other ecosystems adjacent to the dry forest. However, the small extent and fragmented limits of the Equatorial SDF, coupled with the land-use change that affects this ecoregion, represent a risk that, in the case of climate change, these species face a reduction of suitable habitat (Nowakowski et al. 2017), even if currently they also occur in protected areas. This emphasizes the need for the species with a narrow distribution to be targeted for urgent monitoring and conservation measures (Sodhi et al 2008).

### Conservation aspects

The current loss of biodiversity in the study area is the synergic result of a multitude of factors, the most important being habitat loss, fragmentation, pollution, introduction of alien species and unsustainable use of resources (Ceballos and Ortega-Baes 2011). The Equatorial SDF is under severe anthropic pressure (Jara-Guerrero et al. 2019), experiencing a dramatic loss in area in the quality of these forests, exacerbating the biodiversity losses that occurred during the last century, mainly because of agricultural and urban expansion (Mittermeier et al. 1999; Sierra 2013). Originally, 35% of coastal Ecuador was naturally covered with Equatorial SDF, but this ecosystem was reduced to less than 2% by the 1990s (Dodson and Gentry 1991). This alarming situation has catalyzed an effort to protect the last remnants and isolated patches of tropical dry forest (Gentry 1977; Parker and Carr 1992; Best and Kessler 1995; Espinosa et al. 2012; Sierra 2013; Tapia-Armijos et al. 2015). Estimated yearly rate of deforestation in the area was on average of 1.6% between 2000 and 2010 (Sierra 2013). Making matters worse is the fact that the remnants are highly fragmented, reducing their potential of regeneration (Tapia-Armijos et al. 2015). In this context, there is an urgent need for future research evaluating the efficiency of protected areas for the conservation of Equatorial SDF amphibians, under different scenarios of global change.

The level of protection for Equatorial SDF is extremely low (Rivas et al. 2020), less than 5% of its territory being included in nationally protected areas in Ecuador and Peru (Escribano-Avila et al. 2017). To alleviate this aspect, several private entities and local communities are taking steps forward to protect key areas in the region (Escribano-Avila et al. 2017). However, the conservation of this and other ecosystems cannot and should not be the exclusive responsibility of NGOs. The governments of Ecuador and Peru, the civil society of each country (including universities and research centers), and the international community must become more involved in these processes. An essential part of this support is providing the correct information and analysis regarding species distribution, ecology, and status of conservation.

## Supplementary materials

Supplementary material 1 Tables S1, S2 Data type species data Explanation note List of ecosystem types included in the Equatorial Seasonally Dry Forest, based on MAE 2013 and MINAM 2019. Presence of amphibian species in the provinces (Ecuador) or departments (Peru) throughout the Equatorial Seasonally Dry Forest. https://binary.pensoft.net/file/599956This dataset is made available under the Open Database License (http://opendatacommons.org/licenses/odbl/1.0/). The Open Database License (ODbL) is a license agreement intended to allow users to freely share, modify, and use this Dataset while maintaining this same freedom for others, provided that the original source and author(s) are credited.Diego Armijos-Ojeda, Diana Székely, Paul Székely, Dan Cogălniceanu, Diego F. Cisneros-Heredia, Leonardo Ordóñez-Delgado, Adrián Escudero, Carlos Iván Espinosa

Supplementary material 2 Appendix 1. Reference list for life-history characteristics of amphibians of the Equatorial Seasonally Dry Forest (Table 1) Data type reference list https://binary.pensoft.net/file/599957This dataset is made available under the Open Database License (http://opendatacommons.org/licenses/odbl/1.0/). The Open Database License (ODbL) is a license agreement intended to allow users to freely share, modify, and use this Dataset while maintaining this same freedom for others, provided that the original source and author(s) are credited.Diego Armijos-Ojeda, Diana Székely, Paul Székely, Dan Cogălniceanu, Diego F. Cisneros-Heredia, Leonardo Ordóñez-Delgado, Adrián Escudero, Carlos Iván Espinosa

Supplementary material 3 Dataset including amphibian species occurence information, museum specimen numbers, source of data Data type species data https://binary.pensoft.net/file/599958This dataset is made available under the Open Database License (http://opendatacommons.org/licenses/odbl/1.0/). The Open Database License (ODbL) is a license agreement intended to allow users to freely share, modify, and use this Dataset while maintaining this same freedom for others, provided that the original source and author(s) are credited.Diego Armijos-Ojeda, Diana Székely, Paul Székely, Dan Cogălniceanu, Diego F. Cisneros-Heredia, Leonardo Ordóñez-Delgado, Adrián Escudero, Carlos Iván Espinosa
