# Supplementary material for: Amphibians of the equatorial seasonally dry forests of Ecuador and Peru
Source: Zookeys. 2021 Oct 18;1063:23–48. doi: 10.3897/zookeys.1063.69580 (PMC8545738; doi:10.3897/zookeys.1063.69580)
Supplement: Supplementary material 1 — Tables S1, S2 [file zookeys-1063-023-s001.docx]

**Supplementary Table 1**. List of ecosystem types included in the Equatorial Seasonally Dry Forest, based on MAE 2013 and MINAM 2019.

| **Ecosystem name** | **Original name** | **Country** |
| --- | --- | --- |
| Jama – Zapotillo Lowland deciduous forest | Bosque deciduo de tierras bajas del Jama – Zapotillo | Ecuador |
| Jama – Zapotillo Lowland deciduous forest and scurb | Bosque bajo y arbustal deciduo de tierras bajas del Jama – Zapotillo | Ecuador |
| Jama – Zapotillo Lowland desert scrub | Arbustal desértico de tierras bajas del Jama – Zapotillo | Ecuador |
| Catamayo – Alamor foothill deciduos forest | Bosque deciduo piemontano del Catamayo – Alamor | Ecuador |
| Catamayo – Alamor foothill semideciduos forest | Bosque semideciduo piemontano del Catamayo – Alamor | Ecuador |
| Coastal deciduous shrubland and grassland | Arbustal deciduo y herbazal de playas del litoral | Ecuador |
| Deciduous forest of the Equatorial Pacific mountain range | Bosque deciduo de cordillera costera del Pacífico Ecuatorial | Ecuador |
| Anthopical | Intervención | Ecuador |
| No data | Sin Información | Ecuador |
| Seasonally dry forest of plains | Bosque estacionalmente seco de llanura | Peru |
| Seasonally dry forest of hills and mountains | Bosque estacionalmente seco de colina y montaña | Peru |
| Riverine seasonally dry forest | Bosque estacionalmente seco ribereño | Peru |
| Algarrobo dry forests | Bosque estacionalmente seco ribereño (Algarrobal) | Peru |
| Xeric shrublands | Matorral xérico | Peru |

**Supplementary Table 2.** Presence of amphibian species in the provinces (Ecuador) or departments (Peru) throughout the Equatorial Seasonally Dry Forest.

|  | **Ecuador** | | | | | | | **Peru** | | |
| --- | --- | --- | --- | --- | --- | --- | --- | --- | --- | --- |
| **Especie** | **Loja** | **El Oro** | **Azuay** | **Guayas** | **Santa Elena** | **Manabí** | **Esmeraldas** | **Lambayeque** | **Piura** | **Tumbes** |
| *Rhinella alata* |  | X | X | X |  |  |  |  |  |  |
| *Rhinella horribilis* | X | X | X | X | X | X | X | X | X | X |
| *Hyalinobatrachium tatayoi* |  |  |  |  |  | X | X |  |  |  |
| *Ceratophrys stolzmanni* | X | X |  | X | X | X |  |  |  | X |
| *Epipedobates anthonyi* | X | X |  | X |  |  |  |  | X | X |
| *Epipedobates machalilla* |  | X |  | X | X | X |  |  |  |  |
| *Hyloxalus elachyhistus* | X |  |  | X |  |  |  | X | X |  |
| *Hyloxalus infraguttatus* | X | X |  | X | X | X |  |  | X |  |
| *Boana pellucens* |  | X | X | X | X | X |  |  |  | X |
| *Boana rosenbergi* |  |  |  | X | X | X |  |  |  |  |
| *Scinax quinquefasciatus* | X | X |  | X | X | X | X |  |  | X |
| *Scinax sugillatus* |  |  |  | X |  |  |  |  |  |  |
| *Scinax tsachila* | X | X |  | X |  |  |  |  |  |  |
| *Smilisca phaeota* |  | X |  | X | X | X |  |  |  |  |
| *Trachycephalus jordani* | X | X |  | X | X | X | X |  | X | X |
| *Trachycephalus quadrangulum* | X | X |  | X | X | X | X |  |  |  |
| *Engystomops guayaco* |  |  |  | X |  |  |  |  |  |  |
| *Engystomops montubio* |  |  |  |  | X | X |  |  |  |  |
| *Engystomops pustulatus* | X | X |  | X | X | X |  | X | X | X |
| *Engystomops puyango* | X | X |  |  |  |  |  |  |  | X |
| *Engystomops randi* | X | X | X | X |  |  |  |  |  | X |
| *Leptodactylus labrosus* | X | X | X | X | X | X | X | X | X | X |
| *Leptodactylus melanonotus* |  |  |  | X |  | X |  |  |  |  |
| *Leptodactylus ventrimaculatus* | X |  |  |  | X | X | X |  | X |  |
| *Barycholos pulcher* | X | X |  | X | X | X |  |  |  |  |
| *Pristimantis achatinus* |  | X | X | X | X | X | X |  | X |  |
| *Pristimantis lymani* | X | X |  |  |  |  |  | X | X | X |
| *Pristimantis subsigillatus* |  | X | X |  |  | X |  |  |  |  |
| *Pristimantis walkeri* |  |  |  |  | X | X |  |  |  |  |
| *Lithobates bwana* | X | X |  | X |  | X |  |  | X | X |
