# Supplementary material for: Amphibians of the equatorial seasonally dry forests of Ecuador and Peru
Source: Zookeys. 2021 Oct 18;1063:23–48. doi: 10.3897/zookeys.1063.69580 (PMC8545738; doi:10.3897/zookeys.1063.69580)
Supplement: Supplementary material 2 — Appendix 1. Reference list for life-history characteristics of amphibians of the Equatorial Seasonally Dry Forest (Table 1) [file zookeys-1063-023-s002.docx]

**Appendix 1.** Reference list for life-history characteristics of amphibians of the Equatorial Seasonally Dry Forest (Table 1).

1. Dos Santos SP, Ibáñez R, Ron SR (2015) Systematics of the *Rhinella margaritifera* complex (Anura, Bufonidae) from western Ecuador and Panama with insights in the biogeography of *Rhinella alata*. ZooKeys 2015: 109–145.
2. Luna-Gómez MI, García A, Santos-Barrera G (2017) Spatial and temporal distribution and microhabitat use of aquatic breeding amphibians (Anura) in a seasonally dry tropical forest in Chamela, Mexico. Revista de Biología Tropical 65: 1082–1094.
3. Ovalle-Pacheco A, Camacho-Rozo C, Arroyo S (2019) Amphibians from Serrania de Las Quinchas, in the mid-Magdalena river valley, Colombia. Check List 15: 387.
4. Zug GR, Zug PB (1979) The marine toad *Bufo marinus*: a natural history resumé of native populations. Smithsonian Contributions to Zoology 284: 1–58.
5. Guayasamin JM, Cisneros-Heredia DF, McDiarmid RW, Peña P, Hutter CR (2020). Glassfrogs of Ecuador: Diversity, evolution, and conservation. Diversity 12: 1–285.
6. Cruz, F. K., Urgiles, V. L., Sánchez-Nivicela, J. C., Siddons, D. C., & Cisneros-Heredia, D. (2017). Southernmost records of *Hyalinobatrachium fleischmanni* (Anura: Centrolenidae). *Check List*, *13*, 67.
7. Fabrezi M, Quinzio SI (2008) Morphological evolution in Ceratophryinae frogs (Anura, Neobatrachia): the effects of heterochronic changes during larval development and metamorphosis. Zoological Journal of the Linnean Society 154: 752–780.
8. Székely D, Denoël M, Székely P, Cogălniceanu D (2017) Pond drying cues and their effects on growth and metamorphosis in a fast developing amphibian. Journal of Zoology 303: 129–135.
9. Székely D, Szekely P, Stanescu F, Cogălniceanu D, Sinsch U (2018) Breed fast, die young: demography of a poorly known fossorial frog from the xeric Neotropics. Salamandra 54: 37–44.
10. Lötters S, Jungfer K, Henkel FW, Schmidt W (2007) Poison frogs. Biology, species and captive husbandry. Edition Chimaira, Frankfurt am Main, Germany, 668 pp.
11. Montenegro P, del Pino EM (2011) La gastrulación de *Epipedobates anthonyi* (Anura: Dendrobatidae). Revista Ecuatoriana de Medicina y Ciencias Biológicas 32: 24–32.
12. Coloma LA (1995) Ecuadorian frogs of the genus *Colostethus* (Anura: Dendrobatidae). Natural History Museum, Kansas, 84 pp.
13. Elinson RP, del Pino EM (2012) Developmental diversity of amphibians. Wiley Interdisciplinary Reviews: Developmental Biology 1: 345–369.
14. Chuquimarca SL (2018) Adaptaciones a la vida terrestre: análisis fisiológico y del desarrollo de branquias embrionarias en *Hyloxalus nexipus* e *Hyloxalus elachyhistus* (Dendrobatidae) y descripción del desarrollo embrionario de tres anuros con desarrollo directo: *Noblella* aff. *heyeri*, *Noblella* sp.(Craugastoridae) y *Osornophryne occidentalis* (Buffonidae). Bachelor's thesis, Pontificia Universidad Católica del Ecuador, Quito, Ecuador, 131 pp.
15. Pazmiño-Otamendi GI (2012) Territorialidad, comportamiento social, reproducción y vocalización de *Hyloxalus infraguttatus* (Anura: Dendrobatidae). Bachelor's thesis, Pontificia Universidad Católica del Ecuador, Quito, Ecuador, 137pp.
16. Sanchez DA (2013) Larval morphology of dart-poison frogs (Anura: Dendrobatoidea: Aromobatidae and Dendrobatidae). Zootaxa 3637: 569–591.
17. Duellman WE (1971) The identities of some Ecuadorian hylid frogs. Herpetologica 27:212–227.
18. Venegas PJ, Siu-Ting K, Jordán JC (2008) Amphibia, Hylidae, *Hypsiboas pellucens*: First country record, Peru. Check List 4: 214–218.
19. Köhler G (2011) Amphibians of Central America. Herpeton, Offenbach, Germany, 379 pp.
20. Ortega-Andrade HM, Tobar-Suárez C, Arellano M (2011) Tamaño poblacional, uso del hábitat y relaciones interespecíficas de *Agalychnis spurrelli* (Anura: Hylidae) en un bosque húmedo tropical remanente del noroccidente de Ecuador. Papéis Avulsos de Zoologia 51: 1–19.
21. Arteaga-Navarro AF, Bustamante MR, Guayasamin JM (2013) The amphibians and reptiles of Mindo. Life in the cloudforest. Universidad Tecnológica Indoamérica, Quito, Ecuador, 257 pp.
22. Chávez Landi PA (2017) Fisiología térmica de un depredador *Dasythemis* sp. (Odonata: Libellulidae) y su presa *Hypsiboas pellucens* (Anura: Hylidae) y sus posibles implicaciones frente al cambio climático. Bachelor's thesis, Pontificia Universidad Católica del Ecuador, Quito, Ecuador, 65pp.
23. Duellman WE (2001) The Hylid frogs of Middle America. Society for the Study of Amphibians and Reptiles, New York, USA.
24. Lynch JD, Arroyo SB (2009) Risks to Colombian amphibian fauna from cultivation of coca (*Erythroxylum coca*): a geographical analysis. Journal of Toxicology and Environmental Health 72: 974–985.
25. Ferrão M, Colatreli O, de Fraga R, Kaefer IL, Moravec J, Lima AP (2016) High species richness of *Scinax* treefrogs (Hylidae) in a threatened Amazonian landscape revealed by an integrative approach. PloS One 11: e0165679.
26. Ron SR, Duellman WE, Caminer MA, Pazmiño D. (2018). Advertisement calls and DNA sequences reveal a new species of *Scinax* (Anura: Hylidae) on the Pacific lowlands of Ecuador. PloS One 13: e0203169.
27. Duellman WE (1973) Descriptions of new hylid frogs from Colombia and Ecuador. Herpetologica 29: 219–227.
28. McDiarmid RW, Altig R (1990) Description of a bufonid and two hylid tadpoles from western Ecuador. Alytes: International Journal of Batrachology 8: 51–60.
29. Savage JM (2002) The amphibians and reptiles of Costa Rica: a herpetofauna between two continents, between two seas. University of Chicago press, Chicago, 954 pp.
30. Cisneros-Heredia, D. F. (2007) Notes on the natural history of the casque-headed treefrog *Trachycephalus jordani* (Stejneger & Test, 1891). Herpetozoa 20(1/2): 92–94.
31. Ron SR, Venegas PJ, Ortega-Andrade HM, Gagliardi-Urrutia LAG, Salerno PE (2016) Systematics of *Ecnomiohyla tuberculosa* with the description of a new species and comments on the taxonomy of *Trachycephalus typhonius* (Anura, Hylidae). ZooKeys 630: 115–154.
32. Ron SR, Toral E, Rivera M, Terán-Valdez A (2010) A new species of *Engystomops* (Anura: Leiuperidae) from southwestern Ecuador. Zootaxa 2606: 25*–*49.
33. Ron SR, Cannatella DC, Coloma LA (2004) Two new species of *Physalaemus* (Anura: Leptodactylidae) from western Ecuador. Herpetologica 60: 261*–*275.
34. Heyer WR (1969) The adaptive ecology of the species groups of the genus *Leptodactylus* (Amphibia, Leptodactylidae). Evolution 23: 421*–*428.
35. De Sá RO, Grant T, Camargo A, Heyer WR, Ponssa ML, Stanley E (2014) Systematics of the neotropical genus *Leptodactylus* Fitzinger, 1826 (Anura: Leptodactylidae): phylogeny, the relevance of non-molecular evidence, and species accounts. South American Journal of Herpetology 9:s1.
36. Cisneros-Heredia, D. F. (2006). Distribution and ecology of the western Ecuador frog Leptodactylus labrosus (Amphibia: Anura: Leptodactylidae). Zoological Research, 27(3), 225–234.
37. Charruau P, Cedeno-Vázquez JR, Köhler G (2015) Amphibians and reptiles. In: Hernández-Arana HA, Vega-Zepeda A, Ruíz-Zárate MA, Falcón-Alvarez LI, López-Adame H, Herrera-Silveira J, Kaster J (Eds) Biodiversity and conservation of the Yucatán Peninsula. Springer, Cham, 257*–*293.
38. Suazo-Ortuño I, Benítez-Malvido J, Marroquín-Páramo J, Soto Y, Siliceo H, Alvarado-Díaz J (2018) Resilience and vulnerability of herpetofaunal functional groups to natural and human disturbances in a tropical dry forest. Forest Ecology and Management 426: 145*–*157.
39. Caramaschi U, Pombal JP (2001) *Barycholos savagei*: a junior synonym of *Paludicola ternetzi*, with notes on development. Journal of Herpetology, 35: 357*–*360.
40. Coloma LA, Ron S, Cisneros-Heredia DF, Almendáriz A (2004) *Barycholos pulcher.* The IUCN Red List of Threatened Species 2004: <https://dx.doi.org/10.2305/IUCN.UK.2004.RLTS.T56327A11461091.en>. Downloaded on 14 June 2020.
41. Lynch JD, Myers CW (1983) Frogs of the *fitzingeri* group of *Eleutherodactylus* in eastern Panama and Chocoan South America (Leptodactylidae). Bulletin of the American Museum of Natural History 175: 481*–*572.
42. Rojas-Rivera A, Cortés-Bedoya S, Gutiérrez-Cárdenas PD, Castellanos JM (2011) *Pristimantis achatinus* (Cachabi robber frog) Parental care and clutch size. Herpetological Review 42: 588*–*589.
43. Lynch JD, Duellman WE (1997) Frogs of the genus *Eleutherodactylus* (Leptodactylidae) in western Ecuador: systematic, ecology, and biogeography. Natural History Museum, Kansas, 236 pp.
44. Duellman WE, Lehr E (2009) Terrestrial-breeding frogs (Strabomantidae) in Peru. Natur und Tier Verlag, Munster, 382 pp.
45. Almendáriz, A. and J. L. Carr. 1992. Amphibians and Reptile List; January-February Trip. Pp. 128–130. In: T. A. Parker III and J. L. Carr (eds.), Status of Forest Remnants in the Cordillera de la Costa and Adjacent Areas of Southwestern Ecuador. Washington, D. C. Conservation International, RAP Working Paper 2.
46. Hillis DM, De Sá R (1988) Phylogeny and taxonomy of the *Rana palmipes* group (Salientia: Ranidae). Herpetological Monographs 2: 1*–*26.
